# Supplementary material for: Challenges and opportunities to computationally deconvolve heterogeneous tissue with varying cell sizes using single-cell RNA-sequencing datasets
Source: Genome Biol. 2023 Dec 14;24:288. doi: 10.1186/s13059-023-03123-4 (PMC10722720; doi:10.1186/s13059-023-03123-4)
Supplement: Supplementary file 1 — Additional file 1: Supplemental Figure 1. Schematic of collecting orthogonal assays from the same tissue block across donors and tissues. [file 13059_2023_3123_MOESM1_ESM.docx]

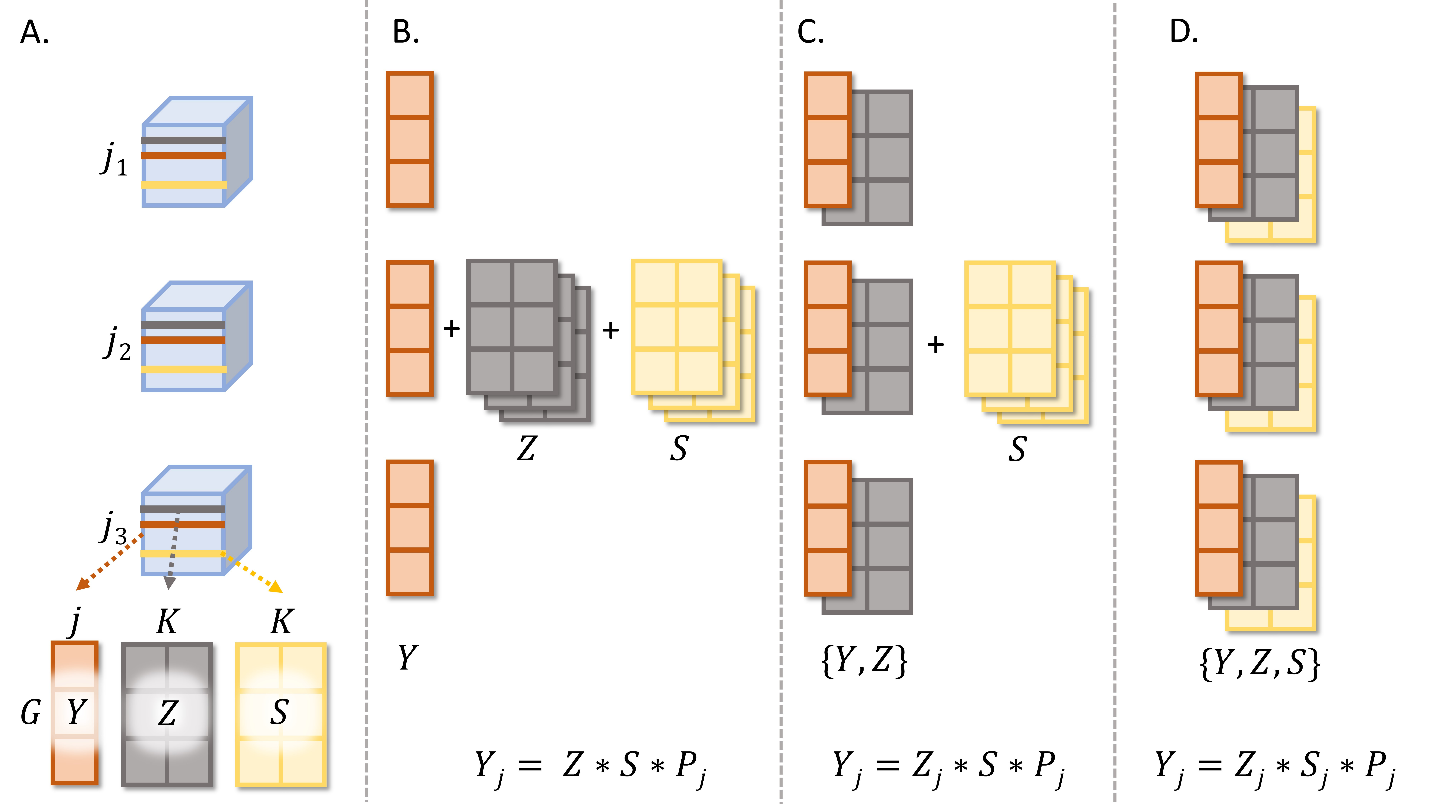


**Supplemental Figure 1. Schematic of collecting orthogonal assays from the same tissue block across donors and tissues. (A)** Tissue block-matched design capturing orthogonal assays from three hypothetical tissue block sections with tissue block labels (j1, j2, j3) where colors indicate (i) a bulk assay Y (shown in red)*,* (ii) cell type reference atlas assay Z (shown in gray)*,* and (iii) cell size scale factors S (shown in yellow). **(B-C)** Three example experimental analysis scenarios, including **(B)** donor-specific bulk analysis, **(C)** donor-specific bulk and cell type reference analysis, and **(D)** donor-specific bulk, cell type reference, and cell sizes analysis. Equations at bottom correspond to each experiment scheme indicating proportional expression of some marker gene g for cell type k across the K cell types for some donor j, where P is the cell type proportion, S is the kth cell size scale factor, and Y is the jth bulk tissue expression level for gene g.
