# Supplementary material for: Challenges and opportunities to computationally deconvolve heterogeneous tissue with varying cell sizes using single-cell RNA-sequencing datasets
Source: Genome Biol. 2023 Dec 14;24:288. doi: 10.1186/s13059-023-03123-4 (PMC10722720; doi:10.1186/s13059-023-03123-4)
Supplement: Supplementary file 2 — Additional file 2. Review history. [file 13059_2023_3123_MOESM2_ESM.docx]

Review History

**First round of review**

**Reviewer 1**

**Comments to author:**

This paper presents a reasonably comprehensive and high-level summary of concerns and "challenges" present in deconvolution algorithms. The specific context of deconvolution methods is reference-based deconvolution using single-cell or single-nucleus RNA sequencing data. The brain tissue is used as a motivating example. The reviewer has the following major concerns.
1. Since the title, abstract and introduction of the paper all elude to "computationally deconvolve heterogeneous tissue", it is disconcerting to the reviewer that none of the six challenges has included sufficient technical (statistical, analytical, computational) details and proper mathematical problem delineation necessary for a general audience. Below is a breakdown of the varying level of technical details for each challenge:
Challenge 1: Good point but what is the "potential confounding of biological variation"? The authors did not introduce this concept (or many other jargons used throughout the paper). Also the reviewer does not agree with the suggested solutions at the end of "Need for measurements of cell type composition from orthogonal platforms", such as using "MERFISH and Xenium data". The proposed solution has not been proven to be valid at any degree.
Challenge 2: A mathematical definition of cell size and how it is used in some existing methods in mathematical models should be provided. The authors are again assuming the readers know exactly what the cell size factor is and why it can be a big problem in deconvolution, hence limiting the target audience to those who already know the issue.
Challenge 3: Here the authors switched to issues with data generation, without providing a mathematical link from the experimental issues back to computational deconvolution, which is the theme of the paper. This link is entirely missing. Also missing or lacking details is a thorough review of existing computational methods that have been attempted to address some of the issues.
Challenge 4: Here the authors switched to some aspects of heuristic decision-making on the single-cell data, which is necessary for using sc/snRNAseq data for deconvolution. This issue is already relatively well-known among everyone who touches single-cell data. Again in this section, there are vague statements that do not quite provide any solutions. The reviewer is further concerned that the literature review for this challenge may not be comprehensive.
Challenge 5: This challenge is trivial, should not be listed in parallel with other challenges.
Challenge 6: Good and straightforward point but there is no in-depth review of the potential solutions proposed so far.
2. The authors stated "new reference datasets featuring multiple orthogonal assays from matched samples have huge potential to shape and inform new studies" as their recommendation. The reviewer is not convinced with this outlook, which is a bit too general anyways. Every newer technology has its new pitfalls. Blindly generating orthogonal assays may lead to a huge waste of computational energy trying to make sense of each assay.
3. Ref 16 (MuSiC) was referred to most frequently as being useful or addressing specific concerns in multiple places of the paper. It is obviously not the only method that has come up with ways to address various challenges. Many methods, including MuSiC, incorporated specific computational steps to address unwanted variations and biases. This goes back to the reviewer's point above that there is a general lack of review of efforts already made to solve aspects of the challenges. This therefore does not represent an unbiased review of the field.

**Reviewer 2**

**Comments to author:**

In this manuscript Maden et al provide a detailed review about the unique challenges when deconvolving transcriptomic datasets obtained from bulk tissues. More specifically, they introduce six specific topics or challenges that cover a wide field of challenges, including in experimental data or design, algorithm development, and biological or technical caveats. In addition, they also make suggestions on how the community could further standardize systematic deconvolution approaches and facilitate more objective comparisons and benchmarking in the future. Their main interest is focused on brain research, although the conceptual limitations and ideas can be generalized to most tissues. The two first challenges, i.e. need for orthogonal measurements and unique cell type characteristics (e.g. size and transcriptional activity), are potentially most novel and interesting and are sprouted directly from recent technology developments (spatial) and observations. Altogether this work is very comprehensive and will be of significant interest to anyone seeking to develop, use or improve existing deconvolution methods. The addition of table 3 (cell type size estimates) is also particularly useful for the community. Nevertheless, I do have a few comments and suggestions that can hopefully further improve the manuscript.

Comments:

1. The authors suggest that it is important to have a single-cell dataset from a matching sample for more accurate deconvolution results. This has indeed been observed by multiple groups in the field. At the same time, they also argue that it is important to build standardized reference atlases, however this is directly in contradiction with the previous suggestion. It would help if they can clarify a little bit more how these seemingly contradicting suggestions can coalesce and what the benefits of both approaches are in different contexts.

2. Although this manuscript is not a benchmarking study it would benefit readers to have some example code to showcase some of the identified challenges. For example, they could deconvolve a brain dataset with different tools and datasets to highlight and visualize some of the challenges they discussed.

3. The authors could consider discussing MAMS (https://www.biorxiv.org/content/10.1101/2023.03.06.531314v1) in challenge 5. It's an effort across institutes, platforms and coding languages to facilitate harmonization and reproducibility of single-cell data.

4. A deconvolution benchmarking paper from 2020 (Cobos et al) evaluated the combined impact from multiple different factors that could affect deconvolution tasks. Most of the tools that they discussed have also been highlighted in this manuscript, except for DWLS, although it consistently ranked as one of the best tools. I would suggest the authors to also look into this tool, since it was specifically designed to work with for rare cell types which is directly related to part of challenge 2 (Cell types vary in abundance, size and total mRNA).

**Authors Response**

**Point-by-point responses to the reviewers’ comments:**

**Response to Reviewer #1**:

***This paper presents a reasonably comprehensive and high-level summary of concerns and "challenges" present in deconvolution algorithms. The specific context of deconvolution methods is reference-based deconvolution using single-cell or single-nucleus RNA sequencing data. The brain tissue is used as a motivating example. The reviewer has the following major concerns.***

We thank the reviewer for the encouraging comments and we hope the concerns and suggestions have been sufficiently addressed below.

***1. Since the title, abstract and introduction of the paper all elude to "computationally deconvolve heterogeneous tissue", it is disconcerting to the reviewer that none of the six challenges has included sufficient technical (statistical, analytical, computational) details and proper mathematical problem delineation necessary for a general audience. Below is a breakdown of the varying level of technical details for each challenge:***

***Challenge 1: Good point but what is the "potential confounding of biological variation"? The authors did not introduce this concept (or many other jargons used throughout the paper). Also the reviewer does not agree with the suggested solutions at the end of "Need for measurements of cell type composition from orthogonal platforms", such as using "MERFISH and Xenium data". The proposed solution has not been proven to be valid at any degree.***

We appreciate the reviewer’s comments here. In Challenge 1, we have now (a) modified and clarified the language around “potential confounding of biological variation” and (b) we have removed the language around offering solutions that have yet to be validated.

Towards the first aim (a), we updated the first paragraph in Challenge 1 with the following (new text in blue):

“When developing a deconvolution method, using matched bulk and single cell/nucleus RNA-seq (sc/snRNA-seq) datasets from the same tissue samples (**Figure 3)** enables controlling for potential variation beyond cell type variation, observed from unwanted factors [(1–4)](https://sciwheel.com/work/citation?ids=3225532,14197382,6860746,15316484&pre=&pre=&pre=&pre=&suf=&suf=&suf=&suf=&sa=0,0,0,0), such as donor-to-donor variation [(5,6)](https://sciwheel.com/work/citation?ids=14087798,8578160&pre=&pre=&suf=&suf=&sa=0,0). For example, confounding variation may come from factors relating to donor demographics (i.e. sex [(3)](https://sciwheel.com/work/citation?ids=6860746&pre=&suf=&sa=0), genetics [(4)](https://sciwheel.com/work/citation?ids=15316484&pre=&suf=&sa=0), diagnosis [(5)](https://sciwheel.com/work/citation?ids=14087798&pre=&suf=&sa=0), etc.), tissue dissection (i.e. tissue microenvironment representation [(6,7)](https://sciwheel.com/work/citation?ids=8578160,14553766&pre=&pre=&suf=&suf=&sa=0,0)), and/or sample quality (i.e. tissue pH, post-mortem interval, RNA quality, etc. [(8–10)](https://sciwheel.com/work/citation?ids=82308,9011192,10438163&pre=&pre=&pre=&suf=&suf=&suf=&sa=0,0,0)), where certain sources need to be evaluated in specific tissues (i.e. expected white and gray matter proportions from brain specimens [(11)](https://sciwheel.com/work/citation?ids=655682&pre=&suf=&sa=0)). Excess variation from such sources can cause challenges downstream to accurately estimate the cell type reference matrix, Z [(7,8)](https://sciwheel.com/work/citation?ids=14553766,82308&pre=&pre=&suf=&suf=&sa=0,0), leading to inaccurate estimates of cell composition, P. This concept is further supported by Wang et al., 2019 [(12)](https://sciwheel.com/work/citation?ids=6313883&pre=&suf=&sa=0), who studied errors from using a sc/snRNA-seq reference dataset from source A to deconvolute a RNA-seq sample from source B and showed these errors can lead to inaccurate estimates of cell composition P for source B, where sources could be distinct donors or studies. Considering the potential limitations of using existing reference atlas datasets, specific sample sourcing schemes, such as generation of multiple orthogonal assays matched to the same tissue block, could alleviate some of these issues. As orthogonal datasets generated from specimens gathered from the same tissue block (a.k.a. “source-matched” samples) are replicates for important clinical and demographic factors, their greater utilization will limit the influence of excess variation of unwanted factors beyond what is possible with modeling strategies alone. We would also advocate the use of these orthogonal matched assays in the development and benchmarking of new algorithms to better evaluate algorithm performance while controlling unwanted confounds such as technical and biological variation.”

We have also updated the last paragraph in Challenge 1 with the following:

“However, orthogonal cell type measures could be extracted from many different data types (**Table 2**), including microscopy images from molecular marker-based protocols such as single molecule fluorescent in situ hybridization (smFISH) [(13)](https://sciwheel.com/work/citation?ids=12290972&pre=&suf=&sa=0). These image-based technologies could offer an opportunity to characterize cell type proportions, as well as other size/shape measurements directly from the tissue. Furthermore, these images can then be integrated with transcriptome-wide gene expression measurements based on emerging spatial transcriptomics technologies [(14–17)](https://sciwheel.com/work/citation?ids=14402183,93458,13741096,13240710&pre=&pre=&pre=&pre=&suf=&suf=&suf=&suf=&sa=0,0,0,0).”

***Challenge 2: A mathematical definition of cell size and how it is used in some existing methods in mathematical models should be provided. The authors are again assuming the readers know exactly what the cell size factor is and why it can be a big problem in deconvolution, hence limiting the target audience to those who already know the issue.***

We thank the reviewer for this suggestion. We have now added a mathematical formulation of deconvolution in the introduction with the following new text:

“With these standard terms, deconvolution is often mathematically described using the equation

*Y = Z * P*, where the goal is to estimate the set of proportions *P* (i.e. where each *p* ∈ *P* satisfies

*0 < p < 1* and *P* sums to *1*). Approaches for estimating *P* have been widely reviewed in the literature

[(18,19)](https://sciwheel.com/work/citation?ids=7659477,4997273&pre=&pre=&suf=&suf=&sa=0,0) and are outside the scope of this review.

In addition, we have now added a mathematical definition of the cell size scale factor to the second subsection of Challenge #2.

“Consider the following standard mathematical formula *Y GxJ = Z GxK * P _KxJ_* with dimensions for *G* marker genes, *J* bulk sample(s), and *K* cell types, which we drop the dimensions after this point for brevity. Assume we have *S_KxK_ = I_KxK_ * s_K_* , where *S* is a matrix, *I_KxK_* is an identity matrix, and *s_K_* is a vector of scalars *_s1...K_* that refer to the size, such as the average mRNA molecules in a cell, for each *kth* cell type, which are often experimentally derived (**Supplemental Figure 1**). Then, the formulation to deconvolute *Y* with cell type scale factors *S* is described as *Y = Z * S * P*, where we can define a new *Z'GxK = Z GxK * S _KxK_* , and then we see a formulation similar to the standard formula as before:

*Y = Z' * P*. It is worth noting that without this transformation, the assumption made by existing deconvolution methods is that cell types are equal sizes, but incorporating this transformation enables models to assume cells have different sizes. Deconvolution accuracies improved when *S* was calculated from a tissue-matched independent reference [(20)](https://sciwheel.com/work/citation?ids=6369672&pre=&suf=&sa=0) and even if cell size estimates were from distinct organisms [(13)](https://sciwheel.com/work/citation?ids=12290972&pre=&suf=&sa=0).”

***Challenge 3: Here the authors switched to issues with data generation, without providing a mathematical link from the experimental issues back to computational deconvolution, which is the theme of the paper. This link is entirely missing. Also missing or lacking details is a thorough review of existing computational methods that have been attempted to address some of the issues.***

We appreciate the reviewer noting this weakness and we have now expanded the text in Challenge 3 with the following text and added a new figure (Supplemental Figure 1). In the first subsection under Challenge 3, we connect the experimental issues to challenges for computational deconvolution with an example of the choice of protocol bias“Similar to donor-to-donor variation leading to unwanted technical variation to estimate the cell type reference matrix Z, sampling RNA from different cellular compartments can also introduce unwanted variation. For example, experimenters can perform “single cell” sequencing by isolating either whole cells (containing both nuclear and cytoplasmic RNA, often performed from fresh tissue) or just the nuclear compartment (containing only nuclear RNA, performed from frozen tissue). While it has been demonstrated that nuclear RNA is representative of RNA from the whole cell [(21,22)](https://sciwheel.com/work/citation?ids=14168103,13678359&pre=&pre=&suf=&suf=&sa=0,0), there can be substantial differences for certain transcripts thereby introducing variability into the data contained in Z.”

Furthermore, we expanded our citations to include a review of known library preparation biases:

“Factors of library bias in RNAseq expression have been well documented and include library prep base composition [(23)](https://sciwheel.com/work/citation?ids=163022&pre=&suf=&sa=0), fragmentation bias [(24)](https://sciwheel.com/work/citation?ids=5389295&pre=&suf=&sa=0), 3’ direction bias [(25)](https://sciwheel.com/work/citation?ids=316534&pre=&suf=&sa=0), and lacking template DNA [(26)](https://sciwheel.com/work/citation?ids=15278162&pre=&suf=&sa=0).”

and existing computational methods that have attempted to address some of these issues:

“A recent benchmark showed library size normalization was crucial for RNAseq deconvolution [(27)](https://sciwheel.com/work/citation?ids=10884275&pre=&suf=&sa=0).

Computational tools [(28–31)](https://sciwheel.com/work/citation?ids=7306046,15278201,17051,5407393&pre=&pre=&pre=&pre=&suf=&suf=&suf=&suf=&sa=0,0,0,0) for correcting library preparation biases include specialized tools for particular bias types, such as DNA sequence-specific bias correction [(32,33)](https://sciwheel.com/work/citation?ids=9107155,12708710&pre=&pre=&suf=&suf=&sa=0,0).”

“Computational methods are available that have been specifically tested across assays [(33–35)](https://sciwheel.com/work/citation?ids=12708710,1435648,1432681&pre=&pre=&pre=&suf=&suf=&suf=&sa=0,0,0), data modalities [(36)](https://sciwheel.com/work/citation?ids=14610570&pre=&suf=&sa=0), and preparation protocols [(37)](https://sciwheel.com/work/citation?ids=531898&pre=&suf=&sa=0). Finally, new analyses of existing datasets can reveal new assay bias sources like cell stress from hypoxia [(38)](https://sciwheel.com/work/citation?ids=12832889&pre=&suf=&sa=0).”

“Computational tools for rare cell type identification have been developed for rare immune cell populations [(39)](https://sciwheel.com/work/citation?ids=59102&pre=&suf=&sa=0), myeloid progenitor cells [(40)](https://sciwheel.com/work/citation?ids=1028522&pre=&suf=&sa=0), and rare brain cells [(41)](https://sciwheel.com/work/citation?ids=627335&pre=&suf=&sa=0). These used a variety of statistical modeling techniques, including latent variable models, such as scLVM [(39)](https://sciwheel.com/work/citation?ids=59102&pre=&suf=&sa=0), and dampened weighted least squares (DWLS) [(42)](https://sciwheel.com/work/citation?ids=7155513&pre=&suf=&sa=0), which outperformed several other methods in a recent benchmark [(43)](https://sciwheel.com/work/citation?ids=10008140&pre=&suf=&sa=0).”

In addition, we have now added Supplemental Figure 1 to illustrate and provide a stronger mathematical link between experimental design and orthogonal assays from the same tissue block across donors and tissues:

**Supplemental Figure 1. Schematic of collecting orthogonal assays from the same tissue block across donors and tissues. (A)** Tissue block-matched design capturing orthogonal assays from three hypothetical tissue block sections with tissue block labels ( _1_ , _2_, _3_) where colors indicate (i) a bulk assay (shown in red)*,*

(ii) cell type reference atlas assay (shown in gray)*,* and (iii) cell size scale factors (shown in yellow). **(B-C)** Three example experimental analysis scenarios, including **(B)** donor-specific bulk analysis, **(C)** donor-specific bulk and cell type reference analysis, and **(D)** donor-specific bulk, cell type reference, and cell sizes analysis. Equations at bottom correspond to each experiment scheme indicating proportional expression of some

marker gene for cell type across the cell types for some donor, where is the cell type proportion, is

the ℎ cell size scale factor, and is the	bulk tissue expression level for gene .

ℎ

***Challenge 4: Here the authors switched to some aspects of heuristic decision-making on the single-cell data, which is necessary for using sc/snRNAseq data for deconvolution. This issue is already relatively well-known among everyone who touches single-cell data. Again in this section, there are vague statements that do not quite provide any solutions. The reviewer is further concerned that the literature review for this challenge may not be comprehensive.***

We appreciate the reviewer’s comment and agree this is an important point. Previous work [(44–46)](https://sciwheel.com/work/citation?ids=11759316,12603601,5027067&pre=&pre=&pre=&suf=&suf=&suf=&sa=0,0,0) demonstrated the choice of cell type annotation and marker selection impacts downstream analyses including clustering, differential expression, and trajectory analyses. Our goal here was to highlight specific decision points that substantially impact downstream deconvolution accuracy. We specifically illustrate and describe how decisions regarding cell type annotation and marker gene selection critically impact the performance of computational deconvolution algorithms. Given feedback from the Reviewer, we have also expanded the literature review around cell type nomenclature and cell type annotation tools:

“Reviews of cell type label management strategies highlight challenges with reconciling types and subtypes [(47)](https://sciwheel.com/work/citation?ids=7142227&pre=&suf=&sa=0) as well as with tracing cell identities to anatomic and spatial regions (Figure 1 in [(36)](https://sciwheel.com/work/citation?ids=14610570&pre=&suf=&sa=0)).

Specialized computational tools automate [(48,49)](https://sciwheel.com/work/citation?ids=10574386,10257867&pre=&pre=&suf=&suf=&sa=0,0) cell type label assignments across data sources, including scType [(50)](https://sciwheel.com/work/citation?ids=12680072&pre=&suf=&sa=0), scAnno [(51)](https://sciwheel.com/work/citation?ids=14834262&pre=&suf=&sa=0), scReClassify [(52)](https://sciwheel.com/work/citation?ids=8171812&pre=&suf=&sa=0), and neural network-based tool NeuCA [(53)](https://sciwheel.com/work/citation?ids=13211439&pre=&suf=&sa=0).”

To the second subsection on choice of the resolution of cell types used (broad versus fine-grain cell types) and their impact on deconvolution algorithms, we added:

“Cell type resolutions are systematically set and benchmarked to understand deconvolution algorithm performances and generalizability [(18)](https://sciwheel.com/work/citation?ids=7659477&pre=&suf=&sa=0). Higher resolutions typically show worse performance [(43)](https://sciwheel.com/work/citation?ids=10008140&pre=&suf=&sa=0), while exclusion of abundant cell types often has a more detrimental impact than exclusion of rare cell types [(20,54)](https://sciwheel.com/work/citation?ids=13028835,6369672&pre=&pre=&suf=&suf=&sa=0,0). Algorithms such as BayesPrism [(55)](https://sciwheel.com/work/citation?ids=12881377&pre=&suf=&sa=0) were designed to handle multiple cell subtypes implicitly and automatically without requiring the user to specify K dimensions for each resolution, and these could be preferred when robust cell type markers are lacking or cell type identity is particularly uncertain or heterogeneous.”

The subsection on the impact of disease (compared to healthy) tissue on detecting cell type marker genes, which are a major input source to deconvolution algorithms, now reads as follows (new text in blue):

“A further consideration for bulk deconvolution methods is heterogeneity introduced by disease state that may influence marker gene expression. As many algorithms are intended for use in bulk tissue samples from disease states, it is important to understand how illness may uniquely impact cell types and their expression of marker genes. Gene expression atlases [(56,57)](https://sciwheel.com/work/citation?ids=4593785,11129215&pre=&pre=&suf=&suf=&sa=0,0) identify cell type marker genes. Development of these atlases parallels technological advances and led to generation of the first whole-organism atlases in an attempt to collect more assays matched to an individual organism than ever before [(56,58)](https://sciwheel.com/work/citation?ids=12393272,4593785&pre=&pre=&suf=&suf=&sa=0,0). Many new tissue- [(56,57,59)](https://sciwheel.com/work/citation?ids=8869441,11129215,4593785&pre=&pre=&pre=&suf=&suf=&suf=&sa=0,0,0) and condition-specific [(60–62)](https://sciwheel.com/work/citation?ids=6887211,14955239,14056475&pre=&pre=&pre=&suf=&suf=&suf=&sa=0,0,0) atlases have been generated from sc/snRNA-seq data that may be limited by differential expression across conditions [(11,63,64)](https://sciwheel.com/work/citation?ids=14087759,655682,6971321&pre=&pre=&pre=&suf=&suf=&suf=&sa=0,0,0). For example, in samples from individuals with Alzheimer’s Disease (AD) relative to neurotypical control subjects, neurons show marker gene repression, while glial cells generally show up-regulation of marker genes [(60)](https://sciwheel.com/work/citation?ids=6887211&pre=&suf=&sa=0). Changes in gene expression have also been reported for psychiatric disorders such as major depression, where prior work showed 16 cell types with altered expression including excitatory and OPC cell types [(65)](https://sciwheel.com/work/citation?ids=8790280&pre=&suf=&sa=0). Computational interfaces [(66)](https://sciwheel.com/work/citation?ids=7695992&pre=&suf=&sa=0) and tools, including scGen [(67)](https://sciwheel.com/work/citation?ids=7244120&pre=&suf=&sa=0), scVI [(68)](https://sciwheel.com/work/citation?ids=6089359&pre=&suf=&sa=0), and scANVI [(69)](https://sciwheel.com/work/citation?ids=10351086&pre=&suf=&sa=0), enable single cell RNA-seq reference use by managing unwanted variation and between-source marker performance and reliability. Further, new tools [(36)](https://sciwheel.com/work/citation?ids=14610570&pre=&suf=&sa=0) and standards [(70)](https://sciwheel.com/work/citation?ids=10851158&pre=&suf=&sa=0) facilitate management of reference atlases from newer technologies measuring combined modalities, such as transcriptomes and proteomes from the same cell. Given that disease-specific differential expression [(5,62)](https://sciwheel.com/work/citation?ids=14087798,14056475&pre=&pre=&suf=&suf=&sa=0,0) can interfere with the effectiveness of cell type signature matrices, cell type marker genes selected for deconvolution should show equivalent expression between healthy and disease conditions. If expression is not equivalent between conditions, further adjustments to either the reference marker or bulk expression data may be necessary and collection of orthogonal matched assays facilitates these efforts (Challenge 1, **Figure 3**). ”

***Challenge 5: This challenge is trivial, should not be listed in parallel with other challenges.***

While we respectfully disagree with the reviewer, we have expanded the text in Challenge 5 to better explain how this challenge can impact the performance of our ability to deconvolute bulk tissues. In the subsection about data-driven cell type labels, we added:

“An individual sc/snRNA-seq experiment can use either manual [(71,72)](https://sciwheel.com/work/citation?ids=12912719,1933801&pre=&pre=&suf=&suf=&sa=0,0), computational [(67–69,73)](https://sciwheel.com/work/citation?ids=14213041,10351086,7244120,6089359&pre=&pre=&pre=&pre=&suf=&suf=&suf=&suf=&sa=0,0,0,0), or

combined [(74)](https://sciwheel.com/work/citation?ids=11093001&pre=&suf=&sa=0) approaches to assign cell type labels or ascertain their abundances (**Table 2**). For certain tissues [(75)](https://sciwheel.com/work/citation?ids=7424478&pre=&suf=&sa=0) and conditions [(61)](https://sciwheel.com/work/citation?ids=14955239&pre=&suf=&sa=0), consulting external references can narrow the cell type or subtype definition according to its expected properties [(59)](https://sciwheel.com/work/citation?ids=8869441&pre=&suf=&sa=0). Despite the availability of existing protocols, recommendations may disagree, protocols may perform suboptimally in a particular experiment, and it may be difficult to reconcile conflicting recommendations. The choice of marker genes impacts downstream analyses [(51,53,67,68)](https://sciwheel.com/work/citation?ids=13211439,14834262,6089359,7244120&pre=&pre=&pre=&pre=&suf=&suf=&suf=&suf=&sa=0,0,0,0). For these reasons, more standard approaches [(76)](https://sciwheel.com/work/citation?ids=14498895&pre=&suf=&sa=0) and flexible analysis strategies are needed.”

We also updated the second subsection of Challenge 5 regarding the value of state-of-the-art data science file formats, which now reads as follows (with new text in blue):

“Publishing key datasets and results with essential documentation using standard data formats is an important part of reproducible computational research [(77–80)](https://sciwheel.com/work/citation?ids=1006581,14742663,9770822,2757582&pre=&pre=&pre=&pre=&suf=&suf=&suf=&suf=&sa=0,0,0,0). Generating, hosting, and distributing large volumes of transcriptomics data and metadata at scale and in a reproducible manner demands substantial time and resources [(58,81)](https://sciwheel.com/work/citation?ids=12393272,12780454&pre=&pre=&suf=&suf=&sa=0,0), and specialized technologies facilitate this effort [(56,82,83)](https://sciwheel.com/work/citation?ids=4593785,2084729,43387&pre=&pre=&pre=&suf=&suf=&suf=&sa=0,0,0). While flat table files (e.g. files with .csv or .tsv extension) are most common, many other data formats allow rapid and memory-efficient access [(84,85)](https://sciwheel.com/work/citation?ids=12380658,13428361&pre=&pre=&suf=&suf=&sa=0,0) to reduce computing time and resources for access and analysis. Some important examples include relational database formats (e.g. structured query language [SQL], hierarchical data format 5 [HDF5]). For example, the active memory required to load expression counts from 77,604 cells and 36,601 genes is 9.32 MB as an HDF5 file using the DelayedArray framework in Bioconductor table versus 22.72 GB as a standard matrix (Data Availability). Specialized data formats are compatible with increasingly used cloud servers and remote computing environments [(86)](https://sciwheel.com/work/citation?ids=4823127&pre=&suf=&sa=0), and large-scale data efforts [(87,88)](https://sciwheel.com/work/citation?ids=12229723,10990536&pre=&pre=&suf=&suf=&sa=0,0) to comprehensively compile and analyze microarray and sequencing data from the Sequence Read Archive [(82)](https://sciwheel.com/work/citation?ids=2084729&pre=&suf=&sa=0), Gene Expression Omnibus [(83)](https://sciwheel.com/work/citation?ids=43387&pre=&suf=&sa=0), Array Express [(89)](https://sciwheel.com/work/citation?ids=6658929&pre=&suf=&sa=0), and other public repositories has led to publication of HDF5-based data compilations and Bioconductor packages. Further, many of the 246 [(90)](https://sciwheel.com/work/citation?ids=15279045&pre=&suf=&sa=0) actively maintained Bioconductor software packages with the SingleCell descriptor use specialized data formats [(91,92)](https://sciwheel.com/work/citation?ids=7878504,111791&pre=&pre=&suf=&suf=&sa=0,0). These include the *SummarizedExperiment* format for most omics data types [(92)](https://sciwheel.com/work/citation?ids=111791&pre=&suf=&sa=0), and the *SingleCellExperiment* format for sc/snRNA-seq expression data [(91,93)](https://sciwheel.com/work/citation?ids=7878504,14739843&pre=&pre=&suf=&suf=&sa=0,0), which is being extended for use with image coordinate information from spatial transcriptomics experiments [(10,94–96)](https://sciwheel.com/work/citation?ids=7346027,10438163,13258710,12907663&pre=&pre=&pre=&pre=&suf=&suf=&suf=&suf=&sa=0,0,0,0). Further, the Azimuth project [(57)](https://sciwheel.com/work/citation?ids=11129215&pre=&suf=&sa=0) provides cell reference datasets for multiple tissues as SeuratObjects [(46)](https://sciwheel.com/work/citation?ids=5027067&pre=&suf=&sa=0), another data class specialized for sc/snRNA-seq data. Newer data formats may be subject to updates that introduce errors or conflicts with other data classes, and resolving data class conflicts frequently demands a high degree of technical knowledge. This is one reason it is important to publish versions along with packages and object classes, in case an older version needs to be used while a newer version is updated. In summary, sequencing data may be published in a variety of formats to facilitate access, methods should include details like versions for computational tools that were used, and researchers should be aware of the many available technologies for reproducible transcriptomics analyses and data sharing.”

***Challenge 6: Good and straightforward point but there is no in-depth review of the potential solutions proposed so far.***

We thank the reviewer for pointing this out. We have now expanded the text and provided a deeper review of potential solutions for Challenge 6. Specifically, we make suggestions for new algorithm developers:

“Deconvolution algorithms (**Table 1**) such as dtangle [(97)](https://sciwheel.com/work/citation?ids=6017166&pre=&suf=&sa=0), SCDC [(98)](https://sciwheel.com/work/citation?ids=8058503&pre=&suf=&sa=0), Bisque [(99)](https://sciwheel.com/work/citation?ids=8798196&pre=&suf=&sa=0), and DWLS [(42)](https://sciwheel.com/work/citation?ids=7155513&pre=&suf=&sa=0) that do not support user-specified cell scale factors could instead use a transformed or rescaled reference (Challenge 2)”

And we also give suggestions for the scenarios where deconvolution is applied across conditions:

“Specific protocols may be consulted to effectively deconvolve specific cell types across conditions, such as for deconvolution of reactive microglia across brain tissue from donors having neuropsychiatric conditions including Alzheimer’s or epilepsy [(45)](https://sciwheel.com/work/citation?ids=12603601&pre=&suf=&sa=0). Marker gene reference atlases across more conditions [(60,61,100)](https://sciwheel.com/work/citation?ids=14955239,6887211,7964670&pre=&pre=&pre=&suf=&suf=&suf=&sa=0,0,0) could also be consulted and utilized, though new standards for systematic cross-condition atlas utilizations may be needed to reconcile expression differences across conditions ([(11,63,64)](https://sciwheel.com/work/citation?ids=14087759,655682,6971321&pre=&pre=&pre=&suf=&suf=&suf=&sa=0,0,0), Challenge 4). Finally, a likelihood-based approach that utilizes confidence intervals for cell proportion predictions [(97)](https://sciwheel.com/work/citation?ids=6017166&pre=&suf=&sa=0) could be extended to quantify prediction uncertainty across tissues and/or tissue donor conditions.”

2. ***The authors stated "new reference datasets featuring multiple orthogonal assays from matched samples have huge potential to shape and inform new studies" as their recommendation. The reviewer is not convinced with this outlook, which is a bit too general anyways. Every newer technology has its new pitfalls. Blindly generating orthogonal assays may lead to a huge waste of computational energy trying to make sense of each assay.***

We thank the reviewer for pointing this out. We did not intend to recommend new and unvalidated technologies. We have clarified this in our Conclusions section:

“Orthogonal assays are important for deconvolution experiments (**Table 2, Supplemental Figure 1**), allow for biological variation to be systematically studied and modeled (Challenge 1), and are more readily managed and analyzed thanks to specialized open-access technologies (Challenge 5).”

3. ***Ref 16 (MuSiC) was referred to most frequently as being useful or addressing specific concerns in multiple places of the paper. It is obviously not the only method that has come up with ways to address various challenges. Many methods, including MuSiC, incorporated specific computational steps to address unwanted variations and biases. This goes back to the reviewer's point above that there is a general lack of review of efforts already made to solve aspects of the challenges. This therefore does not represent an unbiased review of the field.***

We thank the reviewer. We did not intend to provide a biased review of the field, but we agree with the reviewer that our language could be more inclusive of algorithms that use marker variance-based weighting alongside other algorithms not using this approach. We added the following to the second subsection of Challenge 2:

“Similar algorithms using variance-based marker weighting, such as SCDC [(98)](https://sciwheel.com/work/citation?ids=8058503&pre=&suf=&sa=0), do not incorporate cell scale factors, but have been made compatible with outputs from algorithms that do (**Table 1**).”

We also added the following to the second subsection of Challenge 6:

“Deconvolution algorithms (**Table 1**) such as dtangle [(97)](https://sciwheel.com/work/citation?ids=6017166&pre=&suf=&sa=0), SCDC [(98)](https://sciwheel.com/work/citation?ids=8058503&pre=&suf=&sa=0), Bisque [(99)](https://sciwheel.com/work/citation?ids=8798196&pre=&suf=&sa=0), and DWLS [(42)](https://sciwheel.com/work/citation?ids=7155513&pre=&suf=&sa=0) that do not support user-specified cell scale factors could instead use a transformed or rescaled reference (Challenge 2)”

**Response to Reviewer #2**:

***In this manuscript Maden et al provide a detailed review about the unique challenges when deconvolving transcriptomic datasets obtained from bulk tissues. More specifically, they introduce six specific topics or challenges that cover a wide field of challenges, including in experimental data or design, algorithm development, and biological or technical caveats. In addition, they also make suggestions on how the community could further standardize systematic deconvolution approaches and facilitate more objective comparisons and benchmarking in the future. Their main interest is focused on brain research, although the conceptual limitations and ideas can be generalized to most tissues. The two first challenges, i.e. need for orthogonal measurements and unique cell type characteristics (e.g. size and transcriptional activity), are potentially most novel and interesting and are sprouted directly from recent technology developments (spatial) and observations.***

***Altogether this work is very comprehensive and will be of significant interest to anyone seeking to develop, use or improve existing deconvolution methods. The addition of table 3 (cell type size estimates) is also particularly useful for the community. Nevertheless, I do have a few comments and suggestions that can hopefully further improve the manuscript.***

We thank the reviewer for the encouraging comments and we hope the concerns and suggestions have been sufficiently addressed below.

***Comments:***

***1. The authors suggest that it is important to have a single-cell dataset from a matching sample for more accurate deconvolution results. This has indeed been observed by multiple groups in the field. At the same time, they also argue that it is important to build standardized reference atlases, however this is directly in contradiction with the previous suggestion. It would help if they can clarify a little bit more how these seemingly contradicting suggestions can coalesce and what the benefits of both approaches are in different contexts.***

We thank the reviewer for this feedback and now appreciate how our statement suggested contradictory recommendations. To briefly explain, we aimed to call attention to the potential limitations of using existing reference atlas datasets and how specific sample sourcing schemes, such as generation of multiple orthogonal assays matched to the same tissue block, could alleviate some of these issues. While generating sn/scRNA-seq reference data from matched bulk RNA-seq samples would be ideal, we recognize this may not always be feasible due to limitations in tissue availability, funding, or technical expertise. In this case, having robust consensus reference datasets would be a second best option. We would also advocate the use of these orthogonal matched assays in the development and benchmarking of new algorithms to better evaluate algorithm performance while controlling unwanted confounds such as technical and biological variation.

To better clarify these points, we have added the following to Challenge 1:

“Considering the potential limitations of using existing reference atlas datasets, specific sample sourcing schemes, such as generation of multiple orthogonal assays matched to the same tissue block, could alleviate some of these issues. As orthogonal datasets generated from specimens gathered from the same tissue block (a.k.a. “source-matched” samples) are replicates for important clinical and demographic factors, their greater utilization will limit the influence of excess variation of unwanted factors beyond what is possible with modeling strategies alone. We would also advocate the use of these orthogonal matched assays in the development and benchmarking of new algorithms to better evaluate algorithm performance while controlling unwanted confounds such as technical and biological variation.”

We have also revised Challenge 4, which now reads as follows (new text in blue):

“A further consideration for bulk deconvolution methods is heterogeneity introduced by disease state that may influence marker gene expression. As many algorithms are intended for use in bulk tissue samples from disease states, it is important to understand how illness may uniquely impact cell types and their expression of core marker genes. Gene expression atlases [(56,57)](https://sciwheel.com/work/citation?ids=4593785,11129215&pre=&pre=&suf=&suf=&sa=0,0) identify cell type marker genes. Development of these atlases parallels technological advances and led to generation of the first whole-organism atlases in an attempt to collect more assays matched to an individual organism than ever before [(56,58)](https://sciwheel.com/work/citation?ids=12393272,4593785&pre=&pre=&suf=&suf=&sa=0,0). Many new tissue- [(56,57,59)](https://sciwheel.com/work/citation?ids=8869441,11129215,4593785&pre=&pre=&pre=&suf=&suf=&suf=&sa=0,0,0) and condition-specific [(60–62)](https://sciwheel.com/work/citation?ids=6887211,14955239,14056475&pre=&pre=&pre=&suf=&suf=&suf=&sa=0,0,0) atlases have been generated from sc/snRNA-seq data that may be limited by differential expression across conditions [(11,63,64)](https://sciwheel.com/work/citation?ids=14087759,655682,6971321&pre=&pre=&pre=&suf=&suf=&suf=&sa=0,0,0). For example, in samples from individuals with Alzheimer’s Disease (AD) relative to neurotypical control subjects, neurons show marker gene repression, while glial cells generally show up-regulation of marker genes [(60)](https://sciwheel.com/work/citation?ids=6887211&pre=&suf=&sa=0). Changes in gene expression have also been reported for psychiatric disorders such as major depression, where prior work showed 16 cell types with altered expression including excitatory and OPC cell types [(65)](https://sciwheel.com/work/citation?ids=8790280&pre=&suf=&sa=0). Computational interfaces [(66)](https://sciwheel.com/work/citation?ids=7695992&pre=&suf=&sa=0) and tools, including scGen [(67)](https://sciwheel.com/work/citation?ids=7244120&pre=&suf=&sa=0), scVI [(68)](https://sciwheel.com/work/citation?ids=6089359&pre=&suf=&sa=0), and scANVI [(69)](https://sciwheel.com/work/citation?ids=10351086&pre=&suf=&sa=0), enable single cell RNA-seq reference use by managing unwanted variation and between-source marker performance and reliability. Further, new tools [(36)](https://sciwheel.com/work/citation?ids=14610570&pre=&suf=&sa=0) and standards [(70)](https://sciwheel.com/work/citation?ids=10851158&pre=&suf=&sa=0) facilitate management of reference atlases from newer technologies measuring combined modalities, such as transcriptomes and proteomes from the same cell. Given that disease-specific differential expression [(5,62)](https://sciwheel.com/work/citation?ids=14087798,14056475&pre=&pre=&suf=&suf=&sa=0,0) can interfere with the effectiveness of cell type signature matrices, cell type marker genes selected for deconvolution should show equivalent expression between healthy and disease conditions. If expression is not equivalent between conditions, further adjustments to either the reference marker or bulk expression data may be necessary and collection of orthogonal matched assays facilitates these efforts (Challenge 1, **Figure 3**).”

***2. Although this manuscript is not a benchmarking study it would benefit readers to have some example code to showcase some of the identified challenges. For example, they could deconvolve a brain dataset with different tools and datasets to highlight and visualize some of the challenges they discussed.***

We thank the reviewer for this wonderful suggestion. We agree strongly and have been working on a separate benchmark study (unpublished work) that offers two key features (Figure below):

1. In **Rebuttal Figure 1A**, we illustrate a unique dataset that we have generated that includes tissue block-matched snRNA-seq,bulk RNA-seq, and RNA scope data across 10 donors from the DLPFC. The RNA scope imaging data can serve as an orthogonal measurement of the cell type composition of a tissue sample.
2. In **Rebuttal Figure 1B**, we demonstrate the different results produced by two deconvolution algorithms. This is a preliminary result in our evaluation of several deconvolution methods.

We felt these two products warranted their own manuscripts and we plan to preprint the paper in the next year. Until then, we wanted to provide the reviewer with preliminary results. We decided to not include example code in this manuscript and instead focus on the major conceptual issues and challenges surrounding deconvolution.

**Rebuttal Figure 1. Preview of a complementary deconvolution benchmark study A.** Schematic of the experimental design including tissue block-matched snRNA-seq, bulk RNA-seq, and RNA Scope data from the DLPFC across the anterior posterior axis from 10 donors. **B.** Preliminary cell type proportion estimates from two different deconvolution methods, Bisque and MuSiC, across a series of bulk RNA-seq samples, where stacked bar plot colors represent (green) Astrocytes, (pink) EndoMural cells, (purple) Microglial cells, (orange) Oligodendrocytes, (yellow) Oligodendrocyte precursor cells, (Blue) Excitatory neurons, (red) Inhibitory neurons.

3. ***The authors could consider discussing MAMS***

***(***[***https://www.biorxiv.org/content/10.1101/2023.03.06.531314v1)***](https://www.biorxiv.org/content/10.1101/2023.03.06.531314v1)) ***in challenge 5. It's an effort across institutes, platforms and coding languages to facilitate harmonization and reproducibility of single-cell data.***

We thank the reviewer for their suggestion. We now cite the MAMS study [(76)](https://sciwheel.com/work/citation?ids=14498895&pre=&suf=&sa=0) in Challenge #5:

“An individual sc/snRNA-seq experiment can use either manual [(71,72)](https://sciwheel.com/work/citation?ids=12912719,1933801&pre=&pre=&suf=&suf=&sa=0,0), computational [(67–69,73)](https://sciwheel.com/work/citation?ids=14213041,10351086,7244120,6089359&pre=&pre=&pre=&pre=&suf=&suf=&suf=&suf=&sa=0,0,0,0), or combined [(74)](https://sciwheel.com/work/citation?ids=11093001&pre=&suf=&sa=0) approaches to assign cell type labels or ascertain their abundances (**Table 2**). For certain tissues [(75)](https://sciwheel.com/work/citation?ids=7424478&pre=&suf=&sa=0) and conditions [(61)](https://sciwheel.com/work/citation?ids=14955239&pre=&suf=&sa=0), consulting external references can narrow the cell type or subtype definition according to its expected properties [(59)](https://sciwheel.com/work/citation?ids=8869441&pre=&suf=&sa=0). Despite the availability of existing protocols, recommendations may disagree, protocols may perform suboptimally in a particular experiment, and it may be difficult to reconcile conflicting recommendations. The choice of marker genes impacts downstream analyses [(51,53,67,68)](https://sciwheel.com/work/citation?ids=13211439,14834262,6089359,7244120&pre=&pre=&pre=&pre=&suf=&suf=&suf=&suf=&sa=0,0,0,0). For these reasons, more standard approaches [(76)](https://sciwheel.com/work/citation?ids=14498895&pre=&suf=&sa=0) and flexible analysis strategies are needed.”

4. ***A deconvolution benchmarking paper from 2020 (Cobos et al) evaluated the combined impact from multiple different factors that could affect deconvolution tasks. Most of the tools that they discussed have also been highlighted in this manuscript, except for DWLS, although it consistently ranked as one of the best tools. I would suggest the authors to also look into this tool, since it was specifically designed to work with for rare cell types which is directly related to part of challenge 2 (Cell types vary in abundance, size and total mRNA).***

We thank the reviewer for this suggestion. We have added mention of DWLS [(42)](https://sciwheel.com/work/citation?ids=7155513&pre=&suf=&sa=0) in the following new text in Challenge #3:

“Computational tools for rare cell type identification have been developed for rare immune cell populations [(39)](https://sciwheel.com/work/citation?ids=59102&pre=&suf=&sa=0), myeloid progenitor cells [(40)](https://sciwheel.com/work/citation?ids=1028522&pre=&suf=&sa=0), and rare brain cells [(41)](https://sciwheel.com/work/citation?ids=627335&pre=&suf=&sa=0). These used a variety of statistical modeling techniques, including latent variable models, such as scLVM [(39)](https://sciwheel.com/work/citation?ids=59102&pre=&suf=&sa=0), and dampened weighted least squares (DWLS) [(42)](https://sciwheel.com/work/citation?ids=7155513&pre=&suf=&sa=0), which outperformed several other methods in a recent benchmark [(43)](https://sciwheel.com/work/citation?ids=10008140&pre=&suf=&sa=0).”

[Bibliography](https://sciwheel.com/work/bibliography)

1. [Beck TF, Mullikin JC, NISC Comparative Sequencing Program, Biesecker LG. Systematic Evaluation of Sanger Validation of Next-Generation Sequencing Variants. Clin Chem. 2016 Apr;62(4):647–54.](https://sciwheel.com/work/bibliography/3225532)
2. [Arteche-L0pez A, Ávila-Fernández A, Romero R, Riveiro-Álvarez R, L0pez-Martínez MA, Giménez-Pardo A, et al. Sanger sequencing is no longer always necessary based on a single-center validation of 1109 NGS variants in 825 clinical exomes. Sci Rep. 2021 Mar 11;11(1):5697.](https://sciwheel.com/work/bibliography/14197382)
3. [Hagenauer MH, Schulmann A, Li JZ, Vawter MP, Walsh DM, Thompson RC, et al. Inference of cell type content from human brain transcriptomic datasets illuminates the effects of age, manner of death, dissection, and psychiatric diagnosis. PLoS ONE. 2018 Jul 17;13(7):e0200003.](https://sciwheel.com/work/bibliography/6860746)
4. [Doostparast Torshizi A, Duan J, Wang K. A computational method for direct imputation of cell type-specific expression profiles and cellular compositions from bulk-tissue RNA-Seq in brain disorders. NAR Genom Bioinform. 2021 Jun 22;3(2):lqab056.](https://sciwheel.com/work/bibliography/15316484)
5. [Fan J, Lyu Y, Zhang Q, Wang X, Li M, Xiao R. MuSiC2: cell-type deconvolution for multi-condition bulk RNA-seq data. Brief Bioinformatics. 2022 Nov 19;23(6).](https://sciwheel.com/work/bibliography/14087798)
6. [Wang L, Sebra RP, Sfakianos JP, Allette K, Wang W, Yoo S, et al. A reference profile-free](https://sciwheel.com/work/bibliography/8578160)

[deconvolution method to infer cancer cell-intrinsic subtypes and tumor-type-specific stromal profiles. Genome Med. 2020 Feb 28;12(1):24.](https://sciwheel.com/work/bibliography/8578160)

7. [Charytonowicz D, Brody R, Sebra R. Interpretable and context-free deconvolution of multi-scale whole transcriptomic data with UniCell deconvolve. Nat Commun. 2023 Mar 11;14(1):1350.](https://sciwheel.com/work/bibliography/14553766)
8. [Stegle O, Teichmann SA, Marioni JC. Computational and analytical challenges in single-cell transcriptomics. Nat Rev Genet. 2015 Mar;16(3):133–45.](https://sciwheel.com/work/bibliography/82308)
9. [Denisenko E, Guo BB, Jones M, Hou R, de Kock L, Lassmann T, et al. Systematic assessment of tissue dissociation and storage biases in single-cell and single-nucleus RNA-seq workflows. Genome Biol. 2020 Jun 2;21(1):130.](https://sciwheel.com/work/bibliography/9011192)
10. [Maynard KR, Collado-Torres L, Weber LM, Uytingco C, Barry BK, Williams SR, et al. Transcriptome-scale spatial gene expression in the human dorsolateral prefrontal cortex. Nat Neurosci. 2021;24(3):425–36.](https://sciwheel.com/work/bibliography/10438163)
11. [Lipska BK, Deep-Soboslay A, Weickert CS, Hyde TM, Martin CE, Herman MM, et al. Critical factors in gene expression in postmortem human brain: Focus on studies in schizophrenia. Biol Psychiatry. 2006 Sep 15;60(6):650–8.](https://sciwheel.com/work/bibliography/655682)
12. [Wang X, Park J, Susztak K, Zhang NR, Li M. Bulk tissue cell type deconvolution with multi-subject](https://sciwheel.com/work/bibliography/6313883)

[single-cell expression reference. Nat Commun. 2019 Jan 22;10(1):380.](https://sciwheel.com/work/bibliography/6313883)

13. [Sosina OA, Tran MN, Maynard KR, Tao R, Taub MA, Martinowich K, et al. Strategies for cellular deconvolution in human brain RNA sequencing data. F1000Res. 2021 Aug 4;10:750.](https://sciwheel.com/work/bibliography/12290972)
14. [Huuki-Myers L, Spangler A, Eagles N, Montgomery KD, Kwon SH, Guo B, et al. Integrated single cell and unsupervised spatial transcriptomic analysis defines molecular anatomy of the human dorsolateral prefrontal cortex. BioRxiv. 2023 Feb 15;](https://sciwheel.com/work/bibliography/14402183)
15. [Chen KH, Boettiger AN, Moffitt JR, Wang S, Zhuang X. Spatially resolved, highly multiplexed RNA profiling in single cells. Science. 2015 Apr 24;348(6233):aaa6090.](https://sciwheel.com/work/bibliography/93458)
16. [Janesick A, Shelansky R, Gottscho A, Wagner F, Rouault M, Beliakoff G, et al. High resolution mapping of the breast cancer tumor microenvironment using integrated single cell, spatial and in situ analysis of FFPE tissue. BioRxiv. 2022 Oct 7;](https://sciwheel.com/work/bibliography/13741096)
17. [Williams CG, Lee HJ, Asatsuma T, Vento-Tormo R, Haque A. An introduction to spatial transcriptomics for biomedical research. Genome Med. 2022 Jun 27;14(1):68.](https://sciwheel.com/work/bibliography/13240710)
18. [Sturm G, Finotello F, Petitprez F, Zhang JD, Baumbach J, Fridman WH, et al. Comprehensive evaluation of transcriptome-based cell-type quantification methods for immuno-oncology. Bioinformatics. 2019 Jul 15;35(14):i436–45.](https://sciwheel.com/work/bibliography/7659477)
19. [Avila Cobos F, Vandesompele J, Mestdagh P, De Preter K. Computational deconvolution of transcriptomics data from mixed cell populations. Bioinformatics. 2018 Jun 1;34(11):1969–79.](https://sciwheel.com/work/bibliography/4997273)
20. [Monaco G, Lee B, Xu W, Mustafah S, Hwang YY, Carré C, et al. RNA-Seq Signatures Normalized by mRNA Abundance Allow Absolute Deconvolution of Human Immune Cell Types. Cell Rep. 2019 Feb 5;26(6):1627-1640.e7.](https://sciwheel.com/work/bibliography/6369672)
21. [Chamberlin J, Lee Y, Marth G, Quinlan A. Variable RNA sampling biases mediate concordance of single-cell and nucleus sequencing across cell types. BioRxiv. 2022 Aug 3;](https://sciwheel.com/work/bibliography/14168103)
22. [Gupta A, Shamsi F, Altemose N, Dorlhiac GF, Cypess AM, White AP, et al. Characterization of transcript enrichment and detection bias in single-nucleus RNA-seq for mapping of distinct human adipocyte lineages. Genome Res. 2022 Feb;32(2):242–57.](https://sciwheel.com/work/bibliography/13678359)
23. [Aird D, Ross MG, Chen W-S, Danielsson M, Fennell T, Russ C, et al. Analyzing and minimizing PCR amplification bias in Illumina sequencing libraries. Genome Biol. 2011 Feb 21;12(2):R18.](https://sciwheel.com/work/bibliography/163022)
24. [Wery M, Descrimes M, Thermes C, Gautheret D, Morillon A. Zinc-mediated RNA fragmentation allows robust transcript reassembly upon whole transcriptome RNA-Seq. Methods. 2013 Sep 1;63(1):25–31.](https://sciwheel.com/work/bibliography/5389295)
25. [Nagalakshmi U, Wang Z, Waern K, Shou C, Raha D, Gerstein M, et al. The transcriptional landscape of the yeast genome defined by RNA sequencing. Science. 2008 Jun 6;320(5881):1344–9.](https://sciwheel.com/work/bibliography/316534)
26. [Akbari M, Hansen MD, Halgunset J, Skorpen F, Krokan HE. Low copy number DNA template can render polymerase chain reaction error prone in a sequence-dependent manner. J Mol Diagn. 2005 Feb;7(1):36–9.](https://sciwheel.com/work/bibliography/15278162)
27. [Jin H, Liu Z. A benchmark for RNA-seq deconvolution analysis under dynamic testing environments. Genome Biol. 2021 Apr 12;22(1):102.](https://sciwheel.com/work/bibliography/10884275)
28. [Katayama S, Skoog T, Söderhäll C, Einarsdottir E, Krjutškov K, Kere J. Guide for library design and bias correction for large-scale transcriptome studies using highly multiplexed RNAseq methods. BMC Bioinformatics. 2019 Aug 13;20(1):418.](https://sciwheel.com/work/bibliography/7306046)
29. [Tuerk A, Wiktorin G, Güler S. Mixture models reveal multiple positional bias types in RNA-Seq data and lead to accurate transcript concentration estimates. PLoS Comput Biol. 2017 May 15;13(5):e1005515.](https://sciwheel.com/work/bibliography/15278201)
30. [Patro R, Mount SM, Kingsford C. Sailfish enables alignment-free isoform quantification from RNA-seq reads using lightweight algorithms. Nat Biotechnol. 2014 May;32(5):462–4.](https://sciwheel.com/work/bibliography/17051)
31. [Chen L, Zheng S. BCseq: accurate single cell RNA-seq quantification with bias correction. Nucleic Acids Res. 2018 Aug 21;46(14):e82.](https://sciwheel.com/work/bibliography/5407393)
32. [Ni Z, Chen S, Brown J, Kendziorski C. CB2 improves power of cell detection in droplet-based single-cell RNA sequencing data. Genome Biol. 2020 Jun 8;21(1):137.](https://sciwheel.com/work/bibliography/9107155)
33. [Zhang Y-Z, Yamaguchi R, Imoto S, Miyano S. Sequence-specific bias correction for RNA-seq data using recurrent neural networks. BMC Genomics. 2017 Jan 25;18(Suppl 1):1044.](https://sciwheel.com/work/bibliography/12708710)
34. [Zheng W, Chung LM, Zhao H. Bias detection and correction in RNA-Sequencing data. BMC Bioinformatics. 2011 Jul 19;12:290.](https://sciwheel.com/work/bibliography/1435648)
35. [Gao L, Fang Z, Zhang K, Zhi D, Cui X. Length bias correction for RNA-seq data in gene set analyses. Bioinformatics. 2011 Mar 1;27(5):662–9.](https://sciwheel.com/work/bibliography/1432681)
36. [Heumos L, Schaar AC, Lance C, Litinetskaya A, Drost F, Zappia L, et al. Best practices for single-cell analysis across modalities. Nat Rev Genet. 2023 Aug;24(8):550–72.](https://sciwheel.com/work/bibliography/14610570)
37. [Jones DC, Ruzzo WL, Peng X, Katze MG. A new approach to bias correction in RNA-Seq. Bioinformatics. 2012 Apr 1;28(7):921–8.](https://sciwheel.com/work/bibliography/531898)
38. [Ascensión AM, Araúzo-Bravo MJ, Izeta A. The need to reassess single-cell RNA sequencing datasets: the importance of biological sample processing. F1000Res. 2021 Aug 6;10:767.](https://sciwheel.com/work/bibliography/12832889)
39. [Buettner F, Natarajan KN, Casale FP, Proserpio V, Scialdone A, Theis FJ, et al. Computational analysis of cell-to-cell heterogeneity in single-cell RNA-sequencing data reveals hidden subpopulations of cells. Nat Biotechnol. 2015 Feb;33(2):155–60.](https://sciwheel.com/work/bibliography/59102)
40. [Paul F, Arkin Y, Giladi A, Jaitin DA, Kenigsberg E, Keren-Shaul H, et al. Transcriptional heterogeneity and lineage commitment in myeloid progenitors. Cell. 2015 Dec 17;163(7):1663–77.](https://sciwheel.com/work/bibliography/1028522)
41. [Llorens-Bobadilla E, Zhao S, Baser A, Saiz-Castro G, Zwadlo K, Martin-Villalba A. Single-Cell Transcriptomics Reveals a Population of Dormant Neural Stem Cells that Become Activated upon Brain Injury. Cell Stem Cell. 2015 Sep 3;17(3):329–40.](https://sciwheel.com/work/bibliography/627335)
42. [Tsoucas D, Dong R, Chen H, Zhu Q, Guo G, Yuan G-C. Accurate estimation of cell-type composition from gene expression data. Nat Commun. 2019 Jul 5;10(1):2975.](https://sciwheel.com/work/bibliography/7155513)
43. [Avila Cobos F, Alquicira-Hernandez J, Powell JE, Mestdagh P, De Preter K. Benchmarking of cell type deconvolution pipelines for transcriptomics data. Nat Commun. 2020 Nov 6;11(1):5650.](https://sciwheel.com/work/bibliography/10008140)
44. [Tran MN, Maynard KR, Spangler A, Huuki LA, Montgomery KD, Sadashivaiah V, et al. Single-nucleus transcriptome analysis reveals cell-type-specific molecular signatures across reward circuitry in the human brain. Neuron. 2021 Oct 6;109(19):3088-3103.e5.](https://sciwheel.com/work/bibliography/11759316)
45. [Wang M, Song W-M, Ming C, Wang Q, Zhou X, Xu P, et al. Guidelines for bioinformatics of single-cell sequencing data analysis in Alzheimer’s disease: review, recommendation, implementation and application. Mol Neurodegener. 2022 Mar 2;17(1):17.](https://sciwheel.com/work/bibliography/12603601)
46. [Butler A, Hoffman P, Smibert P, Papalexi E, Satija R. Integrating single-cell transcriptomic data across different conditions, technologies, and species. Nat Biotechnol. 2018 Jun;36(5):411–20.](https://sciwheel.com/work/bibliography/5027067)
47. [Diaz-Mejia JJ, Meng EC, Pico AR, MacParland SA, Ketela T, Pugh TJ, et al. Evaluation of methods to assign cell type labels to cell clusters from single-cell RNA-sequencing data. [version 3; peer review: 2 approved, 1 approved with reservations]. F1000Res. 2019 Mar 15;8.](https://sciwheel.com/work/bibliography/7142227)
48. [Pasquini G, Rojo Arias JE, Schäfer P, Busskamp V. Automated methods for cell type annotation on](https://sciwheel.com/work/bibliography/10574386)

[scRNA-seq data. Comput Struct Biotechnol J. 2021 Jan 19;19:961–9.](https://sciwheel.com/work/bibliography/10574386)

49. [Huang Q, Liu Y, Du Y, Garmire LX. Evaluation of Cell Type Annotation R Packages on Single-cell RNA-seq Data. Genomics Proteomics Bioinformatics. 2021 Apr;19(2):267–81.](https://sciwheel.com/work/bibliography/10257867)
50. [Ianevski A, Giri AK, Aittokallio T. Fully-automated and ultra-fast cell-type identification using specific marker combinations from single-cell transcriptomic data. Nat Commun. 2022 Mar 10;13(1):1246.](https://sciwheel.com/work/bibliography/12680072)
51. [Liu H, Li H, Sharma A, Huang W, Pan D, Gu Y, et al. scAnno: a deconvolution strategy-based automatic cell type annotation tool for single-cell RNA-sequencing data sets. Brief Bioinformatics. 2023 May 19;24(3).](https://sciwheel.com/work/bibliography/14834262)
52. [Kim T, Lo K, Geddes TA, Kim HJ, Yang JYH, Yang P. scReClassify: post hoc cell type classification of single-cell rNA-seq data. BMC Genomics. 2019 Dec 24;20(Suppl 9):913.](https://sciwheel.com/work/bibliography/8171812)
53. [Li Z, Feng H. A neural network-based method for exhaustive cell label assignment using single cell RNA-seq data. Sci Rep. 2022 Jan 18;12(1):910.](https://sciwheel.com/work/bibliography/13211439)
54. [Sutton GJ, Poppe D, Simmons RK, Walsh K, Nawaz U, Lister R, et al. Comprehensive evaluation of deconvolution methods for human brain gene expression. Nat Commun. 2022 Mar 15;13(1):1358.](https://sciwheel.com/work/bibliography/13028835)
55. [Chu T, Wang Z, Pe’er D, Danko CG. Cell type and gene expression deconvolution with BayesPrism enables Bayesian integrative analysis across bulk and single-cell RNA sequencing in oncology. Nat Cancer. 2022 Apr 25;3(4):505–17.](https://sciwheel.com/work/bibliography/12881377)
56. [Regev A, Teichmann SA, Lander ES, Amit I, Benoist C, Birney E, et al. The human cell atlas. eLife. 2017 Dec 5;6.](https://sciwheel.com/work/bibliography/4593785)
57. [Hao Y, Hao S, Andersen-Nissen E, Mauck WM, Zheng S, Butler A, et al. Integrated analysis of multimodal single-cell data. Cell. 2021 Jun 24;184(13):3573-3587.](https://sciwheel.com/work/bibliography/11129215)
58. [Quake SR. A decade of molecular cell atlases. Trends Genet. 2022 Aug;38(8):805–10.](https://sciwheel.com/work/bibliography/12393272)
59. [Wang Q, Ding S-L, Li Y, Royall J, Feng D, Lesnar P, et al. The allen mouse brain common coordinate framework: A 3D reference atlas. Cell. 2020 May 14;181(4):936-953.e20.](https://sciwheel.com/work/bibliography/8869441)
60. [Mathys H, Davila-Velderrain J, Peng Z, Gao F, Mohammadi S, Young JZ, et al. Single-cell transcriptomic analysis of Alzheimer’s disease. Nature. 2019 Jun;570(7761):332–7.](https://sciwheel.com/work/bibliography/6887211)
61. [Gabitto MI, Travaglini KJ, Rachleff VM, Kaplan ES, Long B, Ariza J, et al. Integrated multimodal cell atlas of Alzheimer’s disease. BioRxiv. 2023 May 9;](https://sciwheel.com/work/bibliography/14955239)
62. [Rood JE, Maartens A, Hupalowska A, Teichmann SA, Regev A. Impact of the Human Cell Atlas on medicine. Nat Med. 2022 Dec 8;28(12):2486–96.](https://sciwheel.com/work/bibliography/14056475)
63. [Zhu Y, Webster MJ, Murphy CE, Middleton FA, Massa PT, Liu C, et al. Distinct phenotypes of inflammation associated macrophages and microglia in the prefrontal cortex schizophrenia compared to controls. Front Neurosci. 2022 Jun 30;16:858989.](https://sciwheel.com/work/bibliography/14087759)
64. [Velmeshev D, Schirmer L, Jung D, Haeussler M, Perez Y, Mayer S, et al. Single-cell genomics identifies cell type-specific molecular changes in autism. Science. 2019 May 17;364(6441):685–9.](https://sciwheel.com/work/bibliography/6971321)
65. [Nagy C, Maitra M, Tanti A, Suderman M, Théroux J-F, Davoli MA, et al. Single-nucleus transcriptomics of the prefrontal cortex in major depressive disorder implicates oligodendrocyte precursor cells and excitatory neurons. Nat Neurosci. 2020 Jun;23(6):771–81.](https://sciwheel.com/work/bibliography/8790280)
66. [Delaney C, Schnell A, Cammarata LV, Yao-Smith A, Regev A, Kuchroo VK, et al. Combinatorial prediction of marker panels from single-cell transcriptomic data. Mol Syst Biol. 2019 Oct;15(10):e9005.](https://sciwheel.com/work/bibliography/7695992)
67. [Lotfollahi M, Wolf FA, Theis FJ. scGen predicts single-cell perturbation responses. Nat Methods. 2019 Aug;16(8):715–21.](https://sciwheel.com/work/bibliography/7244120)
68. [Lopez R, Regier J, Cole MB, Jordan MI, Yosef N. Deep generative modeling for single-cell transcriptomics. Nat Methods. 2018 Dec;15(12):1053–8.](https://sciwheel.com/work/bibliography/6089359)
69. [Xu C, Lopez R, Mehlman E, Regier J, Jordan MI, Yosef N. Probabilistic harmonization and annotation of single-cell transcriptomics data with deep generative models. Mol Syst Biol. 2021;17(1):e9620.](https://sciwheel.com/work/bibliography/10351086)
70. [Miller JA, Gouwens NW, Tasic B, Collman F, van Velthoven CT, Bakken TE, et al. Common cell type nomenclature for the mammalian brain. eLife. 2020 Dec 29;9.](https://sciwheel.com/work/bibliography/10851158)
71. [Miller BF, Huang F, Atta L, Sahoo A, Fan J. Reference-free cell type deconvolution of multi-cellular pixel-resolution spatially resolved transcriptomics data. Nat Commun. 2022 Apr 29;13(1):2339.](https://sciwheel.com/work/bibliography/12912719)
72. [Li B, Severson E, Pignon J-C, Zhao H, Li T, Novak J, et al. Comprehensive analyses of tumor immunity: implications for cancer immunotherapy. Genome Biol. 2016 Aug 22;17(1):174.](https://sciwheel.com/work/bibliography/1933801)
73. [Christensen E, Luo P, Turinsky A, Husić M, Mahalanabis A, Naidas A, et al. Evaluation of single-cell RNAseq labelling algorithms using cancer datasets. Brief Bioinformatics. 2023 Jan 19;24(1).](https://sciwheel.com/work/bibliography/14213041)
74. [Clarke ZA, Andrews TS, Atif J, Pouyabahar D, Innes BT, MacParland SA, et al. Tutorial: guidelines for annotating single-cell transcriptomic maps using automated and manual methods. Nat Protoc. 2021 Jun;16(6):2749–64.](https://sciwheel.com/work/bibliography/11093001)
75. [Winnubst J, Bas E, Ferreira TA, Wu Z, Economo MN, Edson P, et al. Reconstruction of 1,000 Projection Neurons Reveals New Cell Types and Organization of Long-Range Connectivity in the Mouse Brain. Cell. 2019 Sep 19;179(1):268-281.e13.](https://sciwheel.com/work/bibliography/7424478)
76. [Wang Y, Sarfraz I, Teh WK, Sokolov A, Herb BR, Creasy HH, et al. Matrix and analysis metadata standards (MAMS) to facilitate harmonization and reproducibility of single-cell data. BioRxiv. 2023 Mar 7;](https://sciwheel.com/work/bibliography/14498895)
77. [Peng RD. Reproducible research in computational science. Science. 2011 Dec 2;334(6060):1226–7.](https://sciwheel.com/work/bibliography/1006581)
78. [Bjornson E. Reproducible research: best practices and potential misuse [perspectives]. IEEE Signal Process Mag. 2019 May;36(3):106–23.](https://sciwheel.com/work/bibliography/14742663)
79. [Seirup M, Chu L-F, Sengupta S, Leng N, Browder H, Kapadia K, et al. Reproducibility across single-cell RNA-seq protocols for spatial ordering analysis. PLoS ONE. 2020 Sep 28;15(9):e0239711.](https://sciwheel.com/work/bibliography/9770822)
80. [Piccolo SR, Frampton MB. Tools and techniques for computational reproducibility. Gigascience. 2016 Jul 11;5(1):30.](https://sciwheel.com/work/bibliography/2757582)
81. [Katz K, Shutov O, Lapoint R, Kimelman M, Brister JR, O’Sullivan C. The Sequence Read Archive: a decade more of explosive growth. Nucleic Acids Res. 2022 Jan 7;50(D1):D387–90.](https://sciwheel.com/work/bibliography/12780454)
82. [Leinonen R, Sugawara H, Shumway M, International Nucleotide Sequence Database Collaboration. The sequence read archive. Nucleic Acids Res. 2011 Jan;39(Database issue):D19-21.](https://sciwheel.com/work/bibliography/2084729)
83. [Edgar R, Domrachev M, Lash AE. Gene Expression Omnibus: NCBI gene expression and hybridization array data repository. Nucleic Acids Res. 2002 Jan 1;30(1):207–10.](https://sciwheel.com/work/bibliography/43387)
84. [Palla G, Spitzer H, Klein M, Fischer D, Schaar AC, Kuemmerle LB, et al. Squidpy: a scalable framework for spatial omics analysis. Nat Methods. 2022 Feb;19(2):171–8.](https://sciwheel.com/work/bibliography/12380658)
85. [Hu J, Chen M, Zhou X. Effective and scalable single-cell data alignment with non-linear canonical correlation analysis. Nucleic Acids Res. 2022 Feb 28;50(4):e21.](https://sciwheel.com/work/bibliography/13428361)
86. [Langmead B, Nellore A. Cloud computing for genomic data analysis and collaboration. Nat Rev Genet. 2018 Apr;19(4):208–19.](https://sciwheel.com/work/bibliography/4823127)
87. [Wilks C, Zheng SC, Chen FY, Charles R, Solomon B, Ling JP, et al. recount3: summaries and queries for large-scale RNA-seq expression and splicing. Genome Biol. 2021 Nov 29;22(1):323.](https://sciwheel.com/work/bibliography/12229723)
88. [Maden SK, Thompson RF, Hansen KD, Nellore A. Human methylome variation across Infinium 450K data on the Gene Expression Omnibus. NAR Genom Bioinform. 2021 Jun;3(2):lqab025.](https://sciwheel.com/work/bibliography/10990536)
89. [Athar A, Füllgrabe A, George N, Iqbal H, Huerta L, Ali A, et al. ArrayExpress update - from bulk to single-cell expression data. Nucleic Acids Res. 2019 Jan 8;47(D1):D711–5.](https://sciwheel.com/work/bibliography/6658929)
90. [Bioconductor - BiocViews [Internet]. [cited 2023 Aug 21]. Available from:](https://sciwheel.com/work/bibliography/15279045)
    [https://www.bioconductor.org/packages/devel/BiocViews.html#___Software](https://sciwheel.com/work/bibliography/15279045)
91. [Amezquita RA, Lun ATL, Becht E, Carey VJ, Carpp LN, Geistlinger L, et al. Orchestrating single-cell analysis with Bioconductor. Nat Methods. 2020 Feb;17(2):137–45.](https://sciwheel.com/work/bibliography/7878504)
92. [Huber W, Carey VJ, Gentleman R, Anders S, Carlson M, Carvalho BS, et al. Orchestrating high-throughput genomic analysis with Bioconductor. Nat Methods. 2015 Feb;12(2):115–21.](https://sciwheel.com/work/bibliography/111791)
93. [Davide Risso [Aut C Cph], Michael Cole. scRNAseq. Bioconductor. 2017;](https://sciwheel.com/work/bibliography/14739843)
94. [Hodge RD, Bakken TE, Miller JA, Smith KA, Barkan ER, Graybuck LT, et al. Conserved cell types with divergent features in human versus mouse cortex. Nature. 2019 Sep;573(7772):61–8.](https://sciwheel.com/work/bibliography/7346027)
95. [Pardo B, Spangler A, Weber LM, Page SC, Hicks SC, Jaffe AE, et al. spatialLIBD: an R/Bioconductor package to visualize spatially-resolved transcriptomics data. BMC Genomics. 2022 Jun 10;23(1):434.](https://sciwheel.com/work/bibliography/13258710)
96. [Righelli D, Weber LM, Crowell HL, Pardo B, Collado-Torres L, Ghazanfar S, et al. SpatialExperiment: infrastructure for spatially-resolved transcriptomics data in R using Bioconductor. Bioinformatics. 2022 May 26;38(11):3128–31.](https://sciwheel.com/work/bibliography/12907663)
97. [Hunt GJ, Freytag S, Bahlo M, Gagnon-Bartsch JA. dtangle: accurate and robust cell type deconvolution. Bioinformatics. 2019 Jun 1;35(12):2093–9.](https://sciwheel.com/work/bibliography/6017166)
98. [Dong M, Thennavan A, Urrutia E, Li Y, Perou CM, Zou F, et al. SCDC: bulk gene expression deconvolution by multiple single-cell RNA sequencing references. Brief Bioinformatics. 2020 Jan 10;](https://sciwheel.com/work/bibliography/8058503)
99. [Jew B, Alvarez M, Rahmani E, Miao Z, Ko A, Garske KM, et al. Accurate estimation of cell composition in bulk expression through robust integration of single-cell information. Nat Commun. 2020 Apr 24;11(1):1971.](https://sciwheel.com/work/bibliography/8798196)
100. [Price AJ, Hwang T, Tao R, Burke EE, Rajpurohit A, Shin JH, et al. Characterizing the nuclear and cytoplasmic transcriptomes in developing and mature human cortex uncovers new insight into psychiatric disease gene regulation. Genome Res. 2020 Jan;30(1):1–11.](https://sciwheel.com/work/bibliography/7964670)

**Second round of review**

**Reviewer 1**

The authors have addressed my major concerns. I just have some minor comments remaining.

1.	The fifth subsection of Challenge 3 highlights the systematic differences between bulk and sc/sn RNA-seq experiments, and how these disparities influence deconvolution outcomes. This discussion misses a direct connection to the similar content in Challenge 1, where the authors underscore the significance of matched bulk and sc/snRNA-seq data to mitigate cell type variations.

2.	The text inconsistently uses "deconvolute" in some places and "deconvolve" in others. Please ensure consistent terminology throughout.

**Reviewer 2**

I have no further comments. I applaud the authors for their very well crafted and clear revision of the manuscript. In addition, I look forward to reading their work-in-progress bench-marking paper in the future.
